# Obituary: Marc Leng

**DOI:** 10.1155/MBD.2000.165

**Published:** 2000

**Authors:** Jean-Marc Malinge

**Affiliations:** Centre de Biophysique Moléculaire CNRS Rue Charles Sadron Orléans F-45071 France


### Obituary

### Remembering Marc Leng

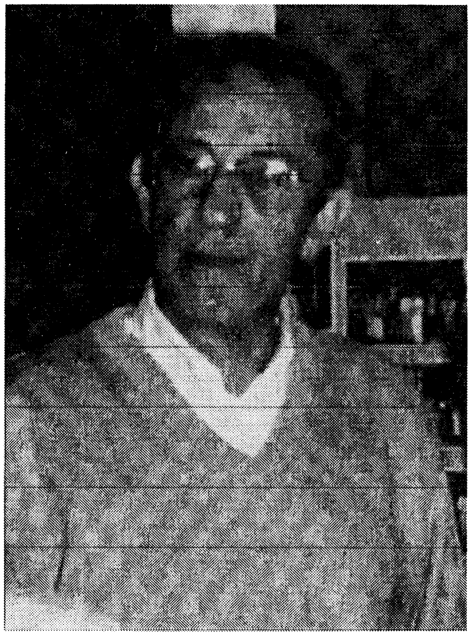

Marc Leng who died on the 7th of May 2000 at the age of 64 from pleural cancer has made an important contribution in the field of Biophysics. Through years of successful studies on deoxyribonucleic acids (DNA) and on the mechanism of action of the well-known anticancer drug cisplatin, he gained high international standing and generated more than 200 publications and 3 patents. His death is a great loss for the scientific community as a whole.

Marc was a pure scientist. Honest, direct, he was a man of his word. He was a hard worker and had a constant intellectual curiosity. Outside scientific matters, he had eclectic tastes from reading to playing tennis or gardening. He was an expert on wine particularly that coming from his childhood region.

Marc Leng was born in Bordeaux in the south west of France in 1935, the son of a medical doctor. He kept a fond memory of his childhood and never totally lost his Bordeaux accent. In 1958, he obtained an engineering diploma in chemistry at the École Nationale Supérieure de Chimie in Bordeaux. Being interested in fundamental research and in macromolecules, he started a PhD in the laboratory of Dr. Benoit at the Centre de Recherche sur les macromolécules (CRM) in Strasbourg. That was the start of a successful career in the CNRS. During these years, he entered the field of polymer chemistry. At the time the CRM had just been created by Pr. Charles Sadron, the French pioneer of polymers science and of French biophysics. The first scientific task of this new research centre was to design and construct apparatus enabling precise characterisation of polymer molecules in dilute solution. Marc's major interest within this area of research was the use of light scattering in the study of synthetic polymers. He graduated from the University of Strasbourg in 1962 and spent the next year and a half fulfilling his military service commitment in the Navy. Following this interlude, he moved to the laboratory of Dr. Garry Felsenfeld as a postdoctoral fellow at the NIH (Bethesda) to pursue his interest in biological macromolecules and particularly in nucleic acids. He began to study DNA on its own or in interaction with polymers of lysine and arginine. It was at that point that he began his lifelong scientific adventure in the DNA world. He discovered that polylysine interacts preferentially with A.T rich DNA. This work was commented on in *Nature* (1969), **223**, 1101 and was qualified as a remarkable finding. During that time working together, Dr. Felsenfeld and Marc became close friends, a relationship that would continue throughout Marc's life and until his last moments.

In 1966, Marc returned in France and he stayed for one year in the laboratory of Dr. Michelson in Paris at the Institut de Biologie Physico-chimique where he pursued his research on polynucleotides. The following year, Charles Sadron founded the Centre de Biophysique Moléculaire in Orléans and invited Marc to continue his research in this new centre and to lead his own team. Very rapidly, he made some major advances. He was the first to show that the ordered form of polyuridilic acid results from the folding of the molecule on itself through base pairing (the hairpin model) and this again was commented on in *Nature* (1970), **227**, 22. With great insight, Marc understood very early that the conformation of macromolecules such as DNA could be as much a part of the storage of genetic information as the genetic code itself. Later discoveries in regulation of gene expression proved how right he was. Indeed, today more and more examples indicate that the plasticity of DNA conformation plays a key role in the interaction with proteins and therefore in gene expression.

In 1975, Marc embarked on a project that was at the time very controversial: the raising of anti-nucleic acids antibodies. Indeed many grant applications he made on this topic were rejected with the following comment: anti-nucleic acids antibodies do not exist. Nevertheless, he persevered and during the next several years, his group worked exclusively on immunisation and the purification and physico-chemical characterisation of these antibodies. One result of these studies was the demonstration that antibodies can be useful tools to detect low amounts of chemical modifications on DNA.

This led Marc to begin studying chemical carcinogenesis, the processes by which normal cells are transformed into cancer cells. Using antibodies raised against DNA that had been modified by some carcinogens, he succeeded in quantifying the amount of adducts formed *in vivo*. In 1983, in collaboration with the Institut Pasteur (Paris), he used these antibodies to develop a genetic immunodiagnostic technique that was patented.

The field of cancer research became a central point in Marc's research. Around 1980, he concentrated his efforts on the study of Z-DNA, the left-handed DNA double helix. He showed that chemical modification of poly(dG-dC).poly(dG-dC) by some carcinogens induces Z-DNA. This led him to investigate the existence of Z-DNA *in vivo* and its possible implication in cancer. Once again, his work was of excellence and he was invited to make opening lectures at international conferences such as the UCLA symposium on Molecular and Cellular Biology at Keystone in 1983.

In 1981, he began his work on the mechanism of action of the anticancer drug cisplatin and new promising platinum based drugs, which was to be his main focus for the next nineteen years. By generating antibodies against platinated DNA he was able to quantify platinum adducts. Subsequently, he studied physico-chemical properties of platinated nucleic acids, the interactions between proteins and platinated DNA and the cross-linking of platinated oligonucleotides to nucleic acids. In addition to his well-known results on distortions in platinated DNA, he discovered that a very fine chemistry of some platinum adducts can be promoted by the double helix i) the formation of new adducts between cisplatin and intercalating drugs and their rearrangements ii) the rearrangement of the transplatin 1,3-intrastrand cross-links into interstrand cross-links. Recently, this latter reaction was used to modulate gene expression as part as an antisense strategy which has been patented. Marc will not be able to pursue this promising research since the very disease he was trying to find a cure for unfortunately defeated him.

Marc was nationally recognised for his scientific contributions. He was appointed director of research of the highest level and had had an important position at the direction of CNRS since 1999. He was co-director of postgraduate studies in Biologie-Biophysique Moléculaire et Cellulaire and lecturer at the University of Orléans-Tours. He has been in charge of 30 theses and Marc's students left his laboratory with a sense of scientific honesty and determination for the future. He was the co-ordinator of the Biomed 2 european contract and participated in the european Cost D8 project "Metal in Medicine". He was also an editorial board member of "Metal-Based Drugs" journal since 1994.

Not everyone's life is as fulfilled as that of Marc Leng's. He had an exceptional career, a joyous marriage, three successful children and he was a loving grandfather. Never since I began my theses with Marc in 1982 did I imagine that his dynamism and his determination could vanish so suddenly. All members of his team as well as friends and colleagues all over the world will never forget his memory.

Jean-Marc Malinge
Centre de Biophysique Moléculaire, CNRS,
Rue Charles Sadron
F-45071 Orléans, France

